# Pneumonia Mortality among Children under 5 in China from 1996 to 2013: An Analysis from National Surveillance System

**DOI:** 10.1371/journal.pone.0133620

**Published:** 2015-07-17

**Authors:** Chunhua He, Leni Kang, Lei Miao, Qi Li, Juan Liang, Xiaohong Li, Yanping Wang, Jun Zhu

**Affiliations:** National Office for Maternal and Child Health Surveillance of China, West China Second University Hospital, Sichuan University, Chengdu, Sichuan, China; London School of Hygiene and Tropical Medicine, UNITED KINGDOM

## Abstract

**Objectives:**

We investigated the mortality rate of pneumonia (PMR) among children under 5 and its time trend from 1996 to 2013 to determine the priorities for ending preventable deaths from pneumonia in children under 5, and share China’s successful experience in reducing PMR with other developing countries.

**Methods:**

We used data from China’s Under 5 Child Mortality Surveillance System (U5CMSS) to calculate the PMR and the proportion of pneumonia deaths to total deaths of children under 5. The data were grouped by urban and rural areas with Cochran-Mantel-Haensel (CMH) test and Chi-square test to examine the differences of PMR and proportion. The time trend was tested by Cochran-Armitage trend test.

**Results:**

The overall PMR of children under 5 was reduced by 85.5% (from 1053.2 to 153.2 per 100,000 live births) from 1996 to 2013, with the urban and rural areas reduced by 69.1% (from 188.4 to 58.2 per 100,000 live births) and 84.7% (from 1252.8 to 191.9 per 100,000 live births), respectively. The overall proportion of pneumonia deaths to total deaths was also declined from 23.4% in 1996 to 12.8% in 2013, with the rural areas decreased from 24.4% to 13.2% and the urban areas decreased from 11.1% to 9.7%. The PMRs in neonates (0-27 days), post-neonates (1-11 months), and childhood (12-59 months) were reduced by 80.7%, 77.4%, and 80.1%, respectively in rural areas, and 71.7%, 69.6%, and 39.0%, respectively in urban areas. During 1996-2013, the PMR in children under 5 years was 4.9 fold higher in rural areas relative to that in urban areas, with relative risk (RR) of 3.6 and 6.4 in neonates and 1- to 59-month-old children, respectively.

**Conclusions:**

PMR in children under 5 significantly declined in China from 1996 to 2013, especially in rural areas. However, huge disparities still existed between rural and urban areas. Infants had the highest PMR, which indicated that interventions aiming at prevention and control of infant pneumonia should be the priority for further reducing PMR in China.

## Introduction

Pneumonia is a preventable and treatable disease. However, it is recognized as “the forgotten killer of children” [1] which kills 1.1–1.4 millions children every year and represents 17–19% of all deaths in children under 5 years of age [2–4]. Mortality due to child pneumonia is strongly associated with malnutrition, poverty and lack of access to quality health care. More than 98% of pneumonia deaths occurred in 68 countries where progress in reducing childhood mortality was slow [5].

In recent years, child pneumonia has attracted the attention of global communities. Nearly 100 organizations/institutions formed the Global Coalition against Child Pneumonia in 2009 aiming at the prevention and control of child pneumonia. The World Health Organization (WHO) and the United Nations Children’s Fund (UNICEF) released the Global Action Plan for Prevention and Control of Pneumonia (GAPP) on the first World Pneumonia Day [6]. GAPP outlines a 6-year plan for the worldwide scale-up of a comprehensive set of interventions to control pneumonia. In 2010, the World Health Assembly passed a resolution recognizing the role of pneumonia as the leading cause of deaths in children, setting out the goal of reducing pneumonia deaths as a global health priority [7].

Pneumonia is also a leading cause of under-five deaths in China. By the end of the 20^th^ century, pneumonia was the first and second leading cause of death among children under 5 in rural and urban areas of China, respectively [8–10]. At the beginning of 1990s, the China’s national mortality rate of pneumonia (PMR) among children under 5 was as high as 1319.2 per 100,000 live births with an estimation of about 350,000 children dying from pneumonia annually, which accounted for 10% of the total pneumonia deaths worldwide [10].

In this article, we described the profile of PMR of children under 5 in China from 1996 to 2013, in order to determine the priorities for ending preventable deaths from child pneumonia, and share China’s successful experience in reducing PMR with other developing countries.

## Methods

### Data source

Data for this study were collected from the database of the China’s Under 5 Child Mortality Surveillance System (U5CMSS). The U5CMSS is a population-based surveillance system with a representative sample of 123 districts/counties (44 urban and 79 rural) across 31 provinces, autonomous regions and municipalities (Excluding Hong Kong, Macau, and Taiwan) during 1996–2006. Since 2007, the sample had been expanded to 336 districts/counties (urban 126 and rural 210). To insure the data quality, we did national training for the additional 213 sites in 2006 before expanding, and conducted provincial and prefectural strengthened trainings in 2007 and 2008. In the meanwhile, we assessed the work quality and data quality according to our quality assessment manual. After that, we included the data in the analysis since 2009. More details about U5CMSS and surveillance subjects have been described in the previous publications [11–13]. This study was approved by the Ethics Committee of West China Second University Hospital, Sichuan University, China.

### Data collection

Village or community doctors were obligated to register and report to township hospitals of all live births and under 5 child deaths within their responsible areas. The doctors of township hospitals or community health service centers were obligated to make inquiries to the child’s family within seven days after receiving the reports, confirm the causes of death, and finish the standard child death registration card. The township hospitals held monthly meeting to check the list of live births and deaths with village/community doctors, and summarize the town data into standard quarterly report forms, then send these data to county Maternal and Child Health (MCH) institutions quarterly. County MCH institutions check and confirm the cases. The reports were sent back to the township hospitals to reinvestigate if errors were found. All the confirmed cases were sent level by level to the county, municipal and provincial MCH institutions for verification and correction, and ultimately submitted to the National Office for Maternal and Child Health Surveillance (NOMCHS) of China for further analysis. (Please see S1 File for the data collection forms and processes.)

### Assessment of causes of death

Doctors from the township hospitals or community health service centers did household surveys for each death in their responsible areas, described the symptoms before death in details on the child death registration card, and assessed the causes of death. If a child died without going to healthcare facilities, the clinical symptoms described in Zhu Futang Practice of Pediatrics were used to determine the pneumonia case: 1) fever; 2) coughing; 3) fast breathing; 4) difficulty breathing; 5) nostrils flare with breathing; or 6) indrawing of the chest [14], using the following algorithm: coughing or difficulty breathing with fast breathing or indrawing of the chest. If a child got diagnoses in healthcare facilities, the causes of death were referred to those diagnoses, usually confirmed by X-ray and other auxiliary examinations.

The medical practitioners at the county or district MCH institutes check all the child death registration cards in their responsible areas quarterly, and confirm the causes of death by reviewing the descriptions in the child death registration cards, medical records or death medical certificates from hospitals. Pediatricians at higher level MCH institutes (i.e., prefectural- or provincial-level) confirm the causes of death according to the information and double check in the quality control. (Please see the S2 File for the process for assessment of causes of death.)

Furthermore, the neonatal death review program was implemented in most surveillance sites before the nationwide implementation in 2009, and the under 5 child death review program was also implemented in most surveillance sites. These programs mainly focused on the assessment of cause of death.

### Quality control

To ensure the accuracy of the data reported, a series of quality controls were conducted regularly and independently at all monitoring levels, including at the township-, county-, municipal-, provincial- and national-level. Quarterly quality control was conducted by township hospitals. MCH institutions at other levels carry out annually quality control in sampling area. The NOMCHS conducted random sampling reviews on 4–8 provinces annually. The reviewing processes included confirmation of birth and death information in coordination with all levels of hospitals, Family Planning Offices, Centers of Disease Control, Public Security Bureau, Civil Affairs Bureau and New Rural Cooperative Medical Service Offices. (Please see the S3 File for the flowchart of quality control.)

### Statistical analysis

Data from 123 and 336 representative sample sites were used for analysis during 1996–2008 and 2009–2013, respectively. The PMR among children under 5 was calculated as number of deaths from pneumonia dividing by number of live births in the same time period, and adjusted with the 3-year moving average under-report rate [11]. The overall PMR between 1996–2003 and 2004–2013 were adjusted based on the 1990 and 2000 census, respectively.

To make a stable estimation, the entire study period of 1996–2013 was further divided into six periods,1996–1998, 1999–2001, 2002–2004, 2005–2007, 2008–2010, and 2011–2013. For each time period, the PMR was adjusted by the national average under-report rate during the corresponding periods.

The Cochran-Armitage trend test was used for analyzing the trends of PMRs and the proportion of pneumonia deaths to total deaths [15]. Poisson regression was used to calculate the annual decline rate of PMR. Cochran-Mantel-Haensel (CMH) test was used for calculating the relative risk (RR) and its 95%CI of PMRs between urban and rural areas [16]. The Chi-Square Test was used for comparing the proportion of PMR to total mortality rate between urban and rural areas. A p value< 0.05 is statistically significant.

## Results

**Fig 1:**
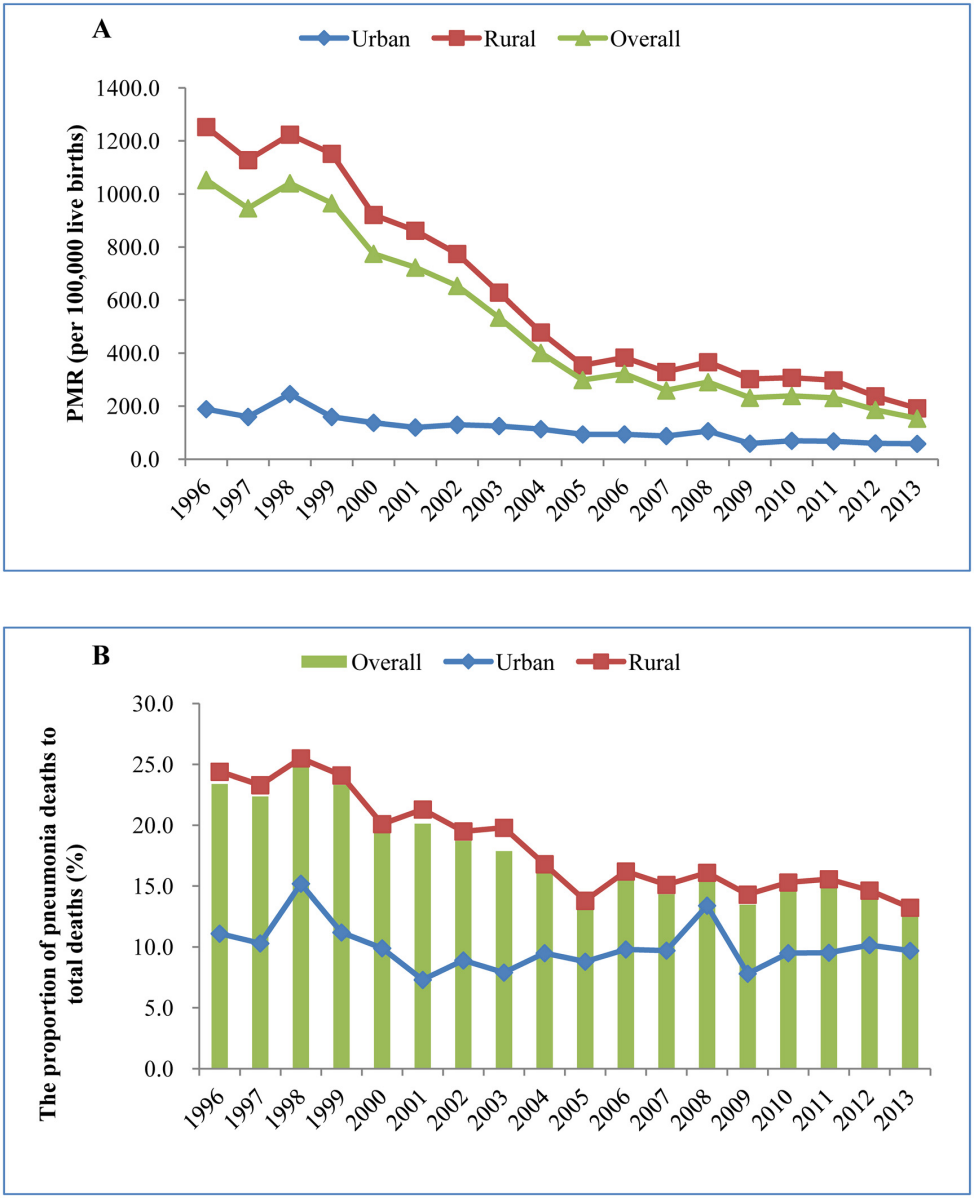
PMR (A) and the proportion of pneumonia deaths to total deaths (B) among children under 5 in China during 1996–2013.

From 1996 to 2013, the overall PMR in children under 5 in China was decreased by 85.5% (from 1053.2 to 153.2 per 100,000 live births; χ^2^ = 3056, *p*_*trend*_<0.001), and the annual rate of decline was 9.2% (95%CI: 8.9%, 9.5%). The PMR significantly decreased from 1996 to 2013 in both urban (decreased by 69.1%, from 188.4 to 58.2 per 100,000 live births) and rural (decreased by 84.7%, from 1252.8 to 191.9 per 100,000 live births) areas among children under 5 (urban: χ^2^ = 283.882, *p*_*trend*_<0.001; rural: χ^2^ = 1599, *p*_*trend*_<0.001). The annual rates of decline were 7.3% (95%CI: 6.4%, 8.1%) and 11.1% (95%CI: 10.5%, 11.6%) in urban and rural areas, respectively (Fig 1A).

The overall proportion of pneumonia deaths to total deaths also reduced by 10.6 percentage point (χ^2^ = 333.309, *p*_*trend*_ <0.001), from 23.4% in 1996 to 12.8% in 2013. In the urban areas, the proportion decreased from 11.1% to 9.7% (χ^2^ = 4.686, *p*_*trend*_ = 0.03), and in the rural areas, it significantly decreased from 24.4% to 13.2% (χ^2^ = 414.508, *p*_*trend*_<0.001) during the study time period (Fig 1B).

**Fig 2:**
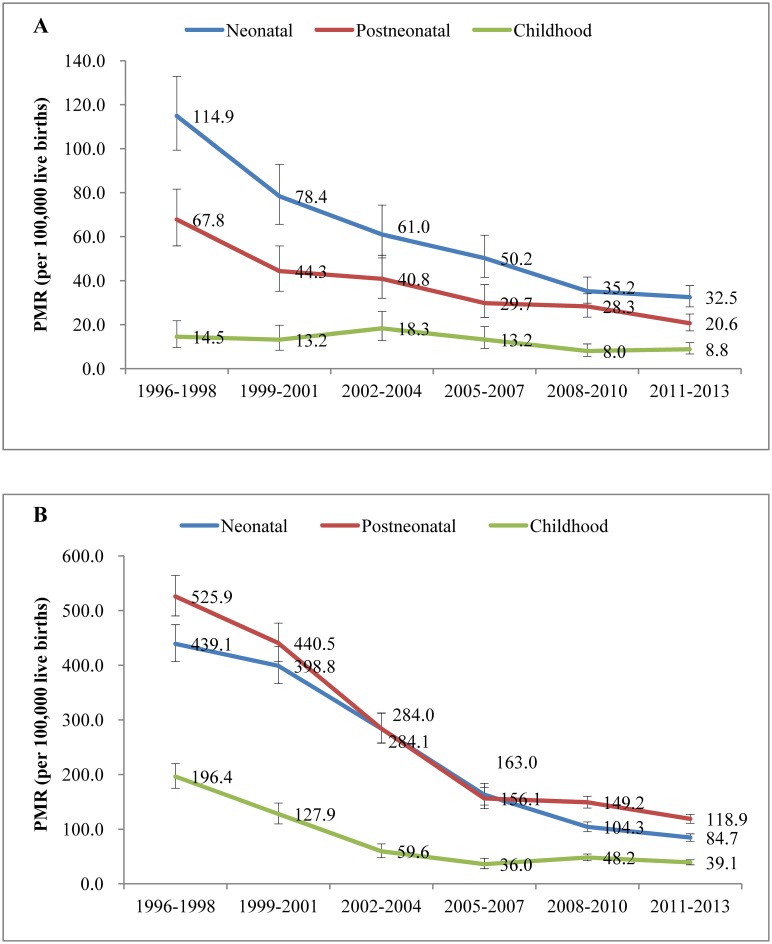
Neonatal, post-neonatal and childhood PMRs in urban (A) and rural areas (B) in China during 1996–2013.

Substantial decreases of PMRs from 1996 to 2013 were observed in all age groups when divided the data into neonates (0-27days), post-neonates (1–11 months), and childhood (12–59 months). In urban areas, the PMRs of the three age groups reduced by 71.7% (χ^2^ = 189.224, *p*_*trend*_<0.001), 69.6% (χ^2^ = 87.084, *p*_*trend*_<0.001), and 39.0% (χ^2^ = 9.375, *p*_*trend*_ = 0.002), respectively (Fig 2A), while in rural areas, the PMRs reduced by 80.7% (χ^2^ = 1541.2, *p*_*trend*_<0.001), 77.4% (χ^2^ = 1391.1, *p*_*trend*_<0.001), and 80.1% (χ^2^ = 460.101, *p*_*trend*_<0.001), respectively in the three age groups (Fig 2B). Neonates had the most dramatic decline of PMRs among the three age groups in both urban and rural areas. The neonatal and post-neonatal PMRs were higher than childhood PMR in both urban and rural areas. During 2011–2013, the neonatal and post-neonatal PMRs were 32.5 and 20.6 per 100,000 live births, respectively in urban areas, and 118.9 and 84.7 per 100,000 live births, respectively in rural areas.

Significant disparities of PMR in children under 5 were observed between rural and urban areas, and in both neonates and children aged 1–59 months (Table 1). During 1996–2013, the PMR in children under 5 years was 4.9 fold higher in rural areas relative to that in urban areas (p<0.001). It was 3.6 and 6.4 fold higher in neonates and children aged 1–59 months (both p<0.001), respectively. Compared with urban areas, the relative risk (RR) of PMR among children under 5 was 6.1 (95%CI: 5.4, 6.9) in rural areas during 1996–1998, and decreased to 3.9 (95%CI: 3.5, 4.4) during 2011–2013. When separated the data into neonates and 1- to 59-month-old children, the RRs also decreased with time, from 3.8 (95%CI: 3.3, 4.5) and 9.1 (95%CI: 7.6, 10.9) in 1996–1998 to 2.6 (95%CI: 2.2, 3.1) and 5.3 (95%CI: 4.5, 6.3), respectively. The gap of PMR between urban and rural areas was the largest during 1999–2001, and started to narrow from 2002–2004. The disparities were also significantly higher in the proportion of pneumonia deaths to total deaths of children under 5 living in rural areas compared to urban areas (*p*<0.001). The proportion was 12.1 and 4.8 percentage points higher in rural areas compared to urban areas in 1996–1998 and 2011–2013, respectively. In general, the proportion decreased with time, however, it changed little from 1999 to 2013 in urban areas.

**Table 1:** The difference of PMR and proportion of pneumonia deaths to total deaths between urban and rural China, 1996–2013.

	Urban			Rural			RR [95% CI]	Comparison of proportion	
	deaths	mortality rate	proportion	deaths	mortality rate	proportion	R to U	χ^2^	P
**Under 5 years**									
1996–1998	314	198.3	12.2	1758	1193.4	24.3	6.1 [5.4, 6.9]	164.487	<.001
1999–2001	225	138.2	9.4	1324	982.2	22.1	7.2 [6.2, 8.2]	182.068	<.001
2002–2004	201	122.1	8.9	900	621.2	18.4	5.1 [4.4, 6.0]	106.916	<.001
2005–2007	197	93.2	9.5	574	355.6	15.0	3.8 [3.3, 4.5]	36.788	<.001
2008–2010	288	74.1	9.8	1516	302.0	15.0	4.1 [3.6, 4.6]	50.952	<.001
2011–2013	331	61.7	9.8	1708	239.9	14.6	3.9 [3.5, 4.4]	51.675	<.001
**Neonates**									
1996–1998	182	114.9	13.5	647	439.1	17.6	3.8 [3.3, 4.5]	12.300	<.001
1999–2001	127	78.4	10.5	538	398.8	18.9	5.1 [4.2, 6.2]	45.194	<.001
2002–2004	101	61.0	9.8	411	284.0	17.3	4.6 [3.7, 5.8]	31.171	<.001
2005–2007	106	50.2	10.3	263	163.0	14.1	3.2 [2.6, 4.1]	8.670	0.003
2008–2010	137	35.2	8.7	523	104.3	12.8	3.0 [2.5, 3.6]	18.039	<.001
2011–2013	175	32.5	9.5	603	84.7	14.4	2.6 [2.2, 3.1]	27.308	<.001
**1–59 months**									
1996–1998	132	83.4	10.9	1111	754.3	31.1	9.1 [7.6, 10.9]	193.051	<.001
1999–2001	97	59.8	8.3	786	583.4	24.9	9.8 [8.0, 12.1]	145.390	<.001
2002–2004	101	61.1	8.2	489	337.2	19.6	5.5 [4.5, 6.8]	78.973	<.001
2005–2007	91	43.0	8.6	311	192.6	15.9	4.5 [3.5, 5.7]	31.163	<.001
2008–2010	151	38.9	11.0	992	197.7	16.4	5.1 [4.3, 6.0]	25.001	<.001
2011–2013	157	29.2	10.1	1105	155.3	14.7	5.3 [4.5, 6.3]	21.831	<.001

## Discussion

China has been progressing substantially in improving child health and has achieved the Millennium Development Goal 4 ahead of the time frame [17]. The mortality rate of children under 5 (U5MR) has been reduced by 70.3% since 1991 [18]. The substantial reduction of U5MR were generally attributable to the collective reduction in infectious diseases, especially pneumonia and diarrhea [11, 18], consistent with what Liu et al have observed in global U5MR [2].

During 1996–2013, the PMR in children under 5 in China was declined by 85.5%. This decline paralleled but exceeded the decline of 73.7% in the overall U5MR, and contributed to 27.3% of the total reduction of U5MR [19, 20]. The decline of PMR can be attributed to the fast economic growth, increasing access to child health care, improvement of child nutrition, and health promotion. During 1996–2013, the average disposable income of urban residents per capita and the average net income of rural residents per capita was increased by 457% and 362%, respectively [19,21]. The proportion of system health management of children under 3 years, an indicator of health care accessibility, increased from 61.4% in 1996 to 87.0% in 2012 [22]. The percentage of moderate malnutrition in children under 5 reduced from 3.73% in 1996 to 1.44% in 2012 [23, 24]. The comprehensive project of maternal and child health supported by the World Bank effectively decreased PMR by implementing case-management of childhood acute respiratory infection (ARI) which was implemented in China during 1995–2001 [25, 26]. The Integrated Management of Childhood Illness (IMCI), which was implemented in 1998 and expanded to 22 provinces by 2003, efficiently promoted the decline of U5MR, especially PMR [27–29]. In order to explore the site-expanding effect, we calculated the PMR with only the original 123 sites, and found that the PMR was lower in the original sites during 2009–2013, although no statistical significance was observed. This can be partly interpreted by the fact that more health promotion programs and measures were firstly implemented in the surveillance sites. (Please see the S1 Fig for the trend analysis for the original 123 sites.)

The proportion of pneumonia deaths to total deaths declined in the entire nation, both in urban and rural areas, consistent with what Williams et al have observed [30]. However, we noticed that the proportion changed little from 1999 to 2013 in urban areas. As we known, the “Four disease management for children” program (i.e., iron deficiency anemia, vitamin D deficiency rickets, pneumonia, and diarrhea) implemented in 1980s, which made the PMR decreased sharply, especially in the urban areas. By the end of 1990s, pneumonia had become the fourth-rank of causes of death among children under 5 years. The proportion of pneumonia deaths to total deaths decreased from 14.6% in 1991–1993 to 11.2% in 1999. Afterwards, more attentions have been paid to other preventable diseases, such as asphyxia, which made the proportion of pneumonia deaths to total deaths changed little.

The PMRs among the three age groups, including neonates, post-neonates, and childhood declined substantially in both urban and rural areas. The decline in the neonates was the most dramatic when compared to the other two age groups. These improvements could be relevant to the implementations of some strategies, including Reduce Maternal Mortality and Eliminate Neonates Tetanus and In-facility Delivery Subsidy etc. These strategies have increased the percentage of system management of pregnancy and in-hospital delivery [24]. Early diagnosis and treatment of maternal infections during pregnancy through prenatal care have successfully reduced the prevalence of neonatal pneumonia. In-hospital delivery has also effectively decreased the incidence and mortality rate of early neonatal pneumonia. The percentage of neonatal family visit conducted by health care personnel increased to 91.8% in 2012 from 81.4% in 1996, also contributed to the decline of the PMR [23, 24].

By decreasing PMR to 153.2 per 100,000 live births in 2013, China has reached the Integrated Global Action Plan for Pneumonia and Diarrhoea (GAPPD)’s goal of overall PMR below 3‰ by 2025 among children under 5. However, due to its large population, China still had 50,000–60,000 annual pneumonia deaths for children under 5 and ranked number 6 among the countries with high numbers of child deaths from pneumonia [31, 32]. China still has higher PMR and the proportion of pneumonia deaths to total deaths in children under 5 than Thailand, Algeria, Belize, Egypt, Sri Lanka, and Peru [32, 33]. The gap between China and developed countries are even greater. China’s urban PMR of 32.5 per 100,000 in 2013 was still higher than that of 17.7 per 100,000 in 1987 in the United States, and that of 5–10 per 100,000 in the 1990s in Switzerland, Denmark, and Japan [34]. Therefore, China is still facing the challenge of ending preventable pneumonia-caused deaths in children by 2025 [35].

Disparities of PMR between rural and urban China still exist. PMR and the proportion of pneumonia deaths to total deaths in rural areas were significantly higher than that of urban areas, in both neonates and 1- to 59-month-old children. This was similar to what Bangladesh et al. had published [36]. The higher PMR was linked to the relatively poorer economy, lower quality of health care, lower levels of maternal education, and lack of accessibility to health care in rural China [37]. The average median net income per capita was ¥24,200 in urban China, but ¥7,907 in rural China in 2013 [21]. The average health care personnel were 3.41 per 1000 people in rural China and 8.54 per 1000 people in urban China in 2012. The average hospital beds in healthcare facilities were 3.11 per 1000 people in rural China and 6.88 per 1000 people in urban China in 2012. In 2008, village and township clinics served as the primary healthcare facility (81.7%) in rural areas, whereas in urban areas, county-level and above hospitals were the primary healthcare facility (50.3%) [24]. Lower maternal education levels impacted the PMR of children by delaying the recognition of symptoms related to pneumonia and hospital visits. The lack of accessibility to health care was also indicated by higher rate of child deaths happened at home in rural areas (57% relative to 23% in urban areas) [38]. Fortunately, the Chinese government has been aware of the regional health care inequality. Its actions to eliminate this inequality has included expanding coverage of the New Rural Cooperative Medical System to improve accessibility of health care, implementing Medicaid for people in poverty, and introducing ARI case-management program and IMCI. These measures were further expanded in 2003. The efficacy of these measures can be seen from the followings: in both neonates and 1- to 59-month-old children, the annual decline rate and reduction of PMR in rural areas were higher than those in urban areas during 1996–2013; the gap of PMR between the rural and urban areas has been narrowed since 2002; during 1996–1998, the PMRs in neonates and 1- to 59-month-old children in rural areas were 3.8 and 9.1 fold of that in urban areas, whereas 2.6 and 5.3 fold during 2011–2013, respectively. This trend was similar to the report of Wang et al [11].

Pneumonia causes more deaths in children under 2 years, particularly infants, due to their immature development of their respiratory and immune systems [4]. During 2011–2013, the PMRs for neonates and post-neonates were 32.5, and 20.6 per 100,000 live births, respectively in urban areas, while 118.9 and 84.7 per 100,000 live births, respectively in rural areas. These were both higher than that of childhood in the same areas. Therefore, interventions aimed at reducing pneumonia in infants should be the priority to ending preventable pneumonia deaths in children.

We acknowledged that there were some limitations in this study. Since we used the data from the nationwide population-based surveillance system, the diagnosis of child pneumonia is a big challenge, especially for the rural areas. However, we did our best to improve the diagnostic accuracy and quality of data. First, there was no information on the etiology diagnosis of death case, which made it impossible to further classify and code the causes of death according to the International Classification of Diseases (ICD). However, the NOMCHS has launched a series of training for staffs working in U5CMSS for death classification and ICD coding. In 2014, the U5CMSS started to use ICD codes for recording the causes of death in order to get more information about child deaths in China and compared with other countries. Second, other diseases or conditions (such as flu, tuberculosis and malaria) may share similar clinical symptoms with pneumonia, which made the diagnosis of pneumonia not so specific. However, most cases were diagnosed in the healthcare facilities confirmed by auxiliary examinations (i.e., X-ray examination). Furthermore, experts would cross check and confirm the causes of death with the support of neonatal and/or under 5 child death evaluation program. Therefore, the possibility of misclassification could be lower. Third, some children died without going to healthcare facilities, and their diagnoses were based on household surveys with child death registration cards, which made the diagnoses more difficult. However, the proportion of children without healthcare facility diagnosis is low (0–3.4% in urban areas and 12.9–16.7% in rural areas from 1996 to 2013). Moreover, NOMCHS started to use the standard verbal autopsy questionnaire recommended by WHO to collect more information for children died without going to healthcare facilities in 2014. It could be more accurate to assess the causes of death. Fourth, with the development of the diagnostic technologies, the accuracy of pneumonia diagnosis increased. This may affect the mortality rate of pneumonia, and we could not estimate the extent. However, we keep using the same definition and conduct a rigorous quality control during the whole study period to lower the influence.

## Conclusions

With the fast economic growth and implementation of governmental policies and strategies in child health, the PMRs among children under 5 have substantially decreased in both urban and rural areas of China from 1996 to 2013. Disparities between rural and urban China were substantially narrowed but still existed. Interventions aimed at reducing pneumonia in infants should be the priority for ending preventable deaths from pneumonia in children under 5.

## Supporting Information

S1 Fig PMR among children under 5 in China during 1996–2013 (for 123 original sites). (TIF)

S1 File Data collection forms and processes. (DOCX)

S2 File Assessment of Causes of Death. (DOCX)

S3 File Flowchart of Quality Control. (DOCX)

## References

[1] UNICEF and WHO. Pneumonia: the forgotten killer of children. New York, UNICEF; 2006.

[2] Liu L, Johnson HL, Cousens S, Perin J, Scott S, Lawn JE, et al Global, regional, and national causes of child mortality: an updated systematic analysis for 2010 with time trends since 2000. Lancet. 2012; 379(9832): 2151–2161. 10.1016/S0140-6736(12)60560-1 22579125

[3] Bhutta ZA, Das JK, Walker N, Rizvi A, Campbell H, Rudan I, et al Interventions to address deaths from childhood pneumonia and diarrhoea equitably: what works and at what cost? Lancet. 2013; 381(9875): 1417–1429. 10.1016/S0140-6736(13)60648-0 23582723

[4] UNICEF, Committing to Child Survival. A Promise Renewed progress report 2013. New York; 2013. Available: http://www.unicef.org/publications/files/APR_Progress_Report_2013_9_Sept_2013.pdf. Accessed 1 November 2013.

[5] UNICEF. Countdown to 2015. Tracking progress in maternal, neonatal and child survival: the 2008 report. New York; 2008.

[6] WHO and UNICEF. Global Action Plan for Prevention and Control of Pneumonia (GAPP). WHO Press; 2009.

[7] Hajjeh R, Whitney CG. Call to action on world pneumonia day. Emerg Infect Dis. 2012; 18(11): 1898–1899. 10.3201/eid1811.121217 23092708 PMC3559175

[8] The cooperative group of the retrospective study on child mortality in 12 provinces. A retrospective study from 12 provinces of China during 1974–1976. The 7th Conference of Chinese Academy of Pediatric. 1978:11. (in Chinese)

[9] Research Group of Pneumonia in MCH Model County. Epidemiologic Surveillance of Pneumonia among Children in the MCH Model Counties in 1986. Chin J Epidemiol. 1990; 11: 257. (in Chinese) 2261612

[10] Liu Y, Lin L, Liu J, Mi J, Liu Q, Cao L. Results of surveillance on death of pneumonia among children under 5 years of age during 1991–1993 in China. Chin J Pediatr. 1996; 34: 365–368. (in Chinese)

[11] Wang YP, Miao L, Dai L, Zhou GX, He CH, Li XH, et al Mortality rate for children under 5 years of age in China from 1996 to 2006. Public health. 2011; 125(5): 301–307. 10.1016/j.puhe.2011.01.003 21524772

[12] Liang J, Mao M, Dai L, Li X, Miao L, Li Q, et al Neonatal mortality due to preterm birth at 28–36 weeks' gestation in China, 2003–2008. Paediatr Perinat Epidemiol. 2011; 25(6): 593–600. 10.1111/j.1365-3016.2011.01232.x 21980948

[13] Wang Y, Zhu J, He C, Li X, Miao L, Liang J. Geographical disparities of infant mortality in rural China. Arch Dis Child Fetal Neonatal Ed. 2012; 97(4): F285–290. 10.1136/archdischild-2011-300412 22247413 PMC3391502

[14] Zhu FT, Wu RP, Hu YM. Zhu Futang Practice of Pediatrics. 1st ed Beijing: People's Medical Publishing House; 1985.

[15] Ruan RS. Epidemiology Principles and Methods, 1st ed Chengdu: Sichuan University Press; 2004.

[16] Agresti A. Categorical Data Analysis. New York: John Wiley and Sons Press; 1990.

[17] Ministry of Foreign Affairs of the People’s Republic of China, United Nations System in China. China’s progress towards the millennium development goals (2013 report). Available: http://www.cn.undp.org/content/dam/china/docs/Publications/UNDP-CH-MDGs2013_english.pdf.

[18] Feng J, Yuan XQ, Zhu J, Li XH, Miao L, He CH, et al Under-5-mortality rate and causes of death in China, 2000 to 2010. Chin J Epidemiol. 2012; 33(6):558–561. (in Chinese) 22883259

[19] National Bureau of Statistics of the People’s Republic of China. China Statistical Yearbook 1997. Beijing: China Statistics Press; 1997 (in Chinese). Available: http://www.stats.gov.cn/tjsj/ndsj/information/nj97/B041A.END

[20] National health and family planning commission in China. China health and family planning statistical digest 2014. Peking Union Medical College Press; 2014. (in Chinese)

[21] National Bureau of Statistics of the People’s Republic of China. China Statistical Yearbook 2014. Beijing: China Statistics Press; 2014. (in Chinese)

[22] National Bureau of Statistics of the People’s Republic of China. China Statistical Yearbook 2013. Beijing: China Statistics Press; 2013.

[23] National health and family planning commission in China. China health Statistical Yearbook; 2008. (in Chinese) Available: http://wsb.moh.gov.cn/htmlfiles/zwgkzt/ptjnj/year2008/7.html

[24] National health and family planning commission in China. China health Statistical Yearbook; 2013. (in Chinese). Available: http://www.nhfpc.gov.cn/htmlfiles/zwgkzt/ptjnj/year2013/index2013.html

[25] Wang H, Yan S, Teng H, Wang X, Wu Y. Analysis the effect of maternity and child health services project on reducing the mortality and morbidity rate in the poorest areas in China. Chin J of Maternal and Child Health Care. 2003; 18(7):395–399. (in Chinese)

[26] Dong ZQ. Strengthen the prevention and treatment of respiratory infections in children. Chin J Pediatr. 2000; 38: 597–598. (in Chinese)

[27] Editorial department of Chinese Journal of Reproductive Health. The effectiveness of comprehensive management of child diseases. Chin J Reprod Health. 2008; 19(5): 280. (in Chinese)

[28] Black RE, Cousens S, Johnson HL, Lawn JE, Rudan I, Bassani DG, et al Global, regional, and national causes of child mortality in 2008: a systematic analysis. Lancet. 2010; 375(9730): 1969–1987. 10.1016/S0140-6736(10)60549-1 20466419

[29] Zhang YF, Dai YH, Zhang SM. Health facility survey on basic equipments and drugs in implementation areas of integrated management of childhood illness (IMCI). Chin J Child Health Care. 2007; 15:519. (in Chinese)

[30] Williams BG, Gouws E, Boschi-Pinto C, et al Estimates of world-wide distribution of child deaths from acute respiratory infections. Lancet Infect Dis 2:25–32, 2002 11892493 10.1016/s1473-3099(01)00170-0

[31] Zhang YF, Dai YH, Zhang SY. Research of the early implementation of integrated management of childhood illness (IMCI). Chin J Child Heath Care. 2003; 11: 76–78. (in Chinese)

[32] WHO. World health statistics 2013. Geneva: WHO; 2013 Available: http://apps.who.int/iris/bitstream/10665/81965/1/9789241564588_eng.pdf?ua=1.

[33] Rudan I, O'Brien KL, Nair H, Liu L, Theodoratou E, Qazi S, et al Epidemiology and etiology of childhood pneumonia in 2010: estimates of incidence, severe morbidity, mortality, underlying risk factors and causative pathogens for 192 countries. journal of global health, J Glob Health. 2013; 3(1): 010401. 10.7189/jogh.03.010401 PMC3700032 23826505

[34] WHO. World health statistic annual 1991. Geneva: WHO; 1992.

[35] WHO and UNICEF. Ending preventable child deaths from pneumonia and diarrhoea by 2025. WHO Press; 2013. Available: http://apps.who.int/iris/bitstream/10665/79200/1/9789241505239_eng.pdf?ua=1.

[36] Bangladesh Demographic and Health Survey 2004. National Institute of Population Research and Training. Dhaka/Calverton, MD: Mitra & Associates/ORC Macro; 2005.

[37] Bryce J, Boschi-Pinto C, Shibuya K, et al WHO estimates of the causes of death in children. Lancet 2005; 365: 1147–1152. 15794969 10.1016/S0140-6736(05)71877-8

[38] Rudan I, Chan KY, Zhang JS, Theodoratou E, Feng XL, Salomon JA, et al Causes of deaths in children younger than 5 years in China in 2008. Lancet. 2010; 375(9720): 1083–1089. 10.1016/S0140-6736(10)60060-8 20346815

