# Supplementary figures and images for: Pneumonia Mortality among Children under 5 in China from 1996 to 2013: An Analysis from National Surveillance System
Source: PLoS One. 2015 Jul 17;10(7):e0133620. doi: 10.1371/journal.pone.0133620 (PMC4505855; doi:10.1371/journal.pone.0133620)

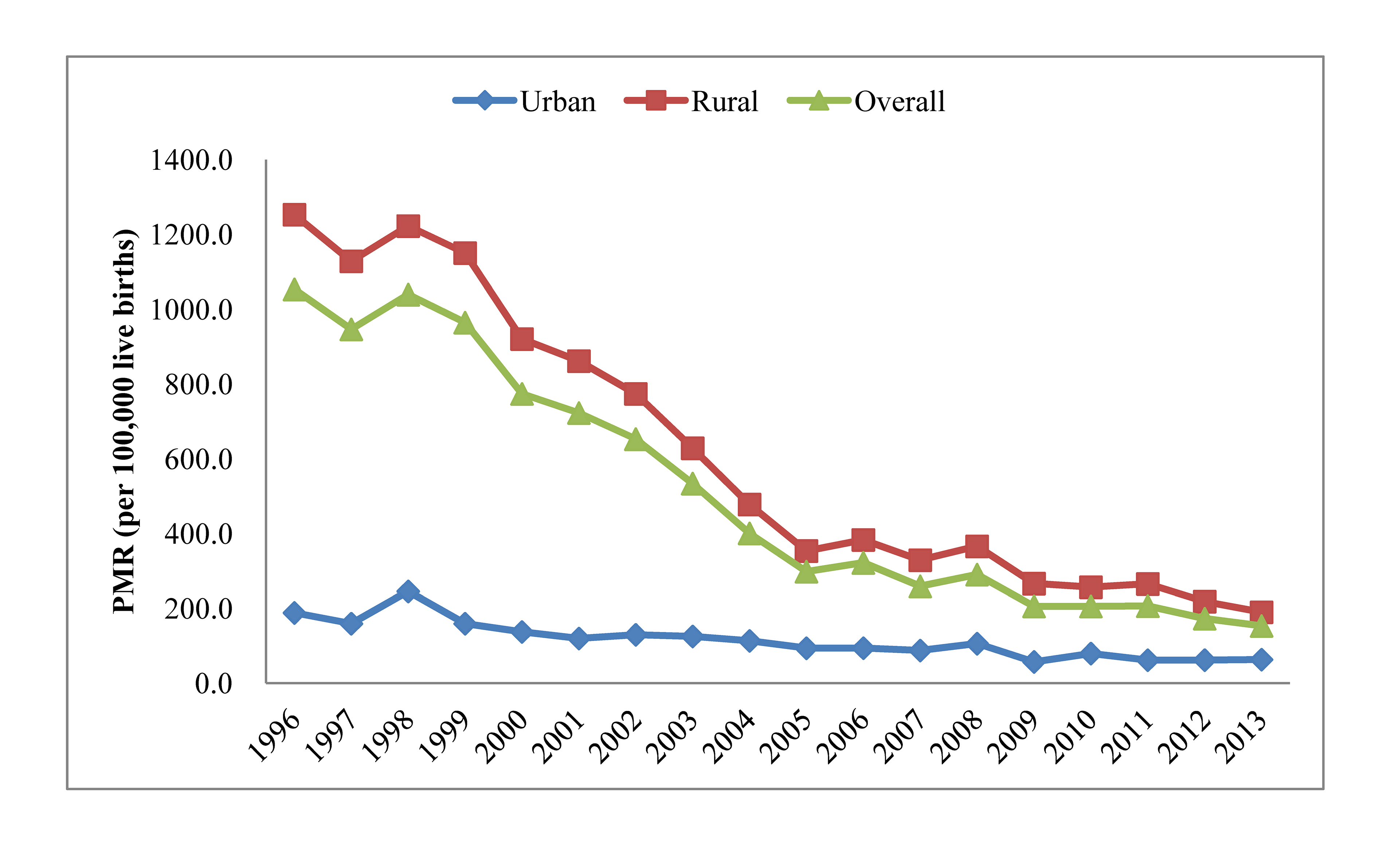

Supplement: S1 Fig — (TIF) [file pone.0133620.s001.tif]
