# Supplementary material for: Pneumonia Mortality among Children under 5 in China from 1996 to 2013: An Analysis from National Surveillance System
Source: PLoS One. 2015 Jul 17;10(7):e0133620. doi: 10.1371/journal.pone.0133620 (PMC4505855; doi:10.1371/journal.pone.0133620)
Supplement: S1 File — (DOCX) [file pone.0133620.s002.docx]

**Child Death Registration Card**

__________District/County□□□□□□

No. □□□□□□□□

Address:____Township/District____Street/Village

Father’s name:_______ Mother’s name_______

Child’s name:________

(1) Registered permanent residence (2) Non-local registered permanent residence for less than one year (3) Non-local registered permanent residence for more than one year □

Sex: 1.Male 2.Female □

| Year | | Month | | Day | |
| --- | --- | --- | --- | --- | --- |
|  |  |  |  |  |  |

Date of birth:

Birth weight: ______g

(1) measured (2) estimated □

Gestational weeks: ______weeks

Place of birth:

(1) provincial/municipal hospital (2) district/county hospital (3) community/township health center (4) village clinic (5) on the way (6) in home □

| Year | | Month | | Day | |
| --- | --- | --- | --- | --- | --- |
|  |  |  |  |  |  |

Date of death:

Age at death: ___years___months___days

Cause of death:____________________

Classification of cause of death □□

Place of death: (1) hospital/clinic (2) on the way

(3) in home □

Premortality treatment: (1) inpatient treatment

(2) outpatient treatment (3) without treatment

□

Level of hospital: (1) provincial/municipal hospital

(2) district/county hospital

(3) community/township health center

(4) village clinic (5) without treatment □

Reason for untreated: (Single Choice)

(1) financial hardship (2) traffic inconvenience

(3) time limited (4) parents unaware of the serious condition (5) manners and customs

(6) others (specify) □

Basis for diagnosis: (1) pathological autopsy

(2) clinical diagnosis (3) inference □

Basis or evidence to infer the causes of death: (Please describe in details, using the blank on the back.)

Report unit_____________ Name of reporter__________ Date of report___________

**Codes for causes of death**

01 dysentery

02 sepsis

03 measles

04 tuberculosis

05 other infectious and parasitic diseases

06 leukemia

07 other neoplasms

08 meningitis

09 other diseases of the nervous system

10 pneumonia

11 other diseases of the respiratory system

12 diarrhea

13 other diseases of the digestive system

14 congenital heart diseases

15 neural tube defects

16 Down's syndrome

17 other congenital malformations

18 premature delivery or low birth weight

19 birth asphyxia

20 tetanus of newborn

21 scleredema of newborn

22 intracranial hemorrhage

23 other diseases of newborn

24 drowning

25 traffic accident

26 accidental suffocation

27 accidental poisoning

28 accidental falls

29 other accidental injuries

30 endocrine, nutritional and metabolic

diseases

31 diseases of blood and blood-forming

organs

32 diseases of the circulatory system

33 diseases of the urinary system

34 other diseases

35 ill-defined and unknown causes of

death

**List of Under-five Children**

| No. | Father’s name | Mother’s name | Child’s name | Sex | Date of birth | year | | | | year | | | | year | | | | year | | | | Comments |
| --- | --- | --- | --- | --- | --- | --- | --- | --- | --- | --- | --- | --- | --- | --- | --- | --- | --- | --- | --- | --- | --- | --- |
|  |  |  |  |  |  | Q1 | Q1 | Q1 | Q2 | Q3 | Q4 | Q2 | Q3 | Q4 | Q2 | Q3 | Q4 | Q4 | Q2 | Q3 | Q4 |  |
|  |  |  |  |  |  |  |  |  |  |  |  |  |  |  |  |  |  |  |  |  |  |  |
|  |  |  |  |  |  |  |  |  |  |  |  |  |  |  |  |  |  |  |  |  |  |  |
|  |  |  |  |  |  |  |  |  |  |  |  |  |  |  |  |  |  |  |  |  |  |  |
|  |  |  |  |  |  |  |  |  |  |  |  |  |  |  |  |  |  |  |  |  |  |  |
|  |  |  |  |  |  |  |  |  |  |  |  |  |  |  |  |  |  |  |  |  |  |  |
|  |  |  |  |  |  |  |  |  |  |  |  |  |  |  |  |  |  |  |  |  |  |  |
|  |  |  |  |  |  |  |  |  |  |  |  |  |  |  |  |  |  |  |  |  |  |  |
|  |  |  |  |  |  |  |  |  |  |  |  |  |  |  |  |  |  |  |  |  |  |  |
|  |  |  |  |  |  |  |  |  |  |  |  |  |  |  |  |  |  |  |  |  |  |  |
|  |  |  |  |  |  |  |  |  |  |  |  |  |  |  |  |  |  |  |  |  |  |  |
|  |  |  |  |  |  |  |  |  |  |  |  |  |  |  |  |  |  |  |  |  |  |  |
|  |  |  |  |  |  |  |  |  |  |  |  |  |  |  |  |  |  |  |  |  |  |  |
|  |  |  |  |  |  |  |  |  |  |  |  |  |  |  |  |  |  |  |  |  |  |  |
|  |  |  |  |  |  |  |  |  |  |  |  |  |  |  |  |  |  |  |  |  |  |  |
|  |  |  |  |  |  |  |  |  |  |  |  |  |  |  |  |  |  |  |  |  |  |  |
|  |  |  |  |  |  |  |  |  |  |  |  |  |  |  |  |  |  |  |  |  |  |  |

**Quarterly Summary Table for Under-5 Child Death**

20__(Year)___(Quarter)

__________Province/Autonomous Region/Municipality___________District/County

Number of people in the monitoring streets/townships______________ Number of under-5 children_______________________

| Name of the monitoring street/  township | Registered permanent residence | | | | | Non-local registered permanent residence | | | | | | | | | |
| --- | --- | --- | --- | --- | --- | --- | --- | --- | --- | --- | --- | --- | --- | --- | --- |
|  |  |  |  |  |  | Total | | | | | Reside for over one year | | | | |
|  | Live births | | Deaths | | | Live births | | Deaths | | | Live births | | Deaths | | |
|  | Male | Female | 0 year  (neonates) | 0-4 years | 1-4 years | Male | Female | 0 year  (neonates) | 0-4 years | 1-4 years | Male | Female | 0 year  (neonates) | 0-4 years | 1-4 years |
|  |  |  |  |  |  |  |  |  |  |  |  |  |  |  |  |
|  |  |  |  |  |  |  |  |  |  |  |  |  |  |  |  |
|  |  |  |  |  |  |  |  |  |  |  |  |  |  |  |  |
|  |  |  |  |  |  |  |  |  |  |  |  |  |  |  |  |
|  |  |  |  |  |  |  |  |  |  |  |  |  |  |  |  |
|  |  |  |  |  |  |  |  |  |  |  |  |  |  |  |  |
|  |  |  |  |  |  |  |  |  |  |  |  |  |  |  |  |
| Total |  |  |  |  |  |  |  |  |  |  |  |  |  |  |  |

**Data Collection, Reporting Processes for U5CMSS**

Department of women and children’s health, National Health and Family Planning Commission of the People’s Republic of China.

Information issue; Maternal and child health policy decision.

National Office for Maternal and Child Health Surveillance

Data audit, analysis and annual analysis report writing.

Submit the annual analysis report before the next February.

Report quarterly summary table quarterly, and annually summary table, child death registration cards and the surveillance quality survey questionnaire of children's mortality in December.

MCH institutions at province-level

Audit quarterly summary table and transcribe the child death registration card to the list of death register or copy it; Organize neonatal death review program.

Report quarterly summary table quarterly, and annually summary table, child death registration cards and the surveillance quality survey questionnaire of children's mortality in November.

MCH institutions at prefecture-level

Audit quarterly summary table and transcribe the child death registration card to the list of death register or copy it; Organize neonatal death review program.

Report quarterly summary table quarterly, and annually summary table, child death registration cards and the surveillance quality survey questionnaire of children's mortality in October.

MCH institutions at county-level

Summarize the quarterly summary table of each townships, fill in the quarterly summary table of the county, and transcribe the child death registration card to the list of death register; Organize neonatal death review program.

Township hospitals/community health care facilities

Summarize the number of live births of each village, fill in the list of under-five children and quarterly summary table; Do face-to-face household survey for each death, fill in the child death registration card and list of death register.

Report quarterly summary table and child death registration cards quarterly.

Village doctors

Report list of under-five children monthly; and report the death clues timely.

Quality control

Fill in the list of under-five children

Quality control

Quality control

Quality control

Quality control
