# Supplementary material for: Pneumonia Mortality among Children under 5 in China from 1996 to 2013: An Analysis from National Surveillance System
Source: PLoS One. 2015 Jul 17;10(7):e0133620. doi: 10.1371/journal.pone.0133620 (PMC4505855; doi:10.1371/journal.pone.0133620)
Supplement: S2 File — (DOCX) [file pone.0133620.s003.docx]

**Assessment of causes of death**

Check and confirm the causes of death by reviewing the descriptions in the child death registration cards, medical records or death medical certificates.

**Village or community doctors**

Report death clues within 10 days

Household surveys within 7 days

**Township hospitals or community health service centers**

**County- or district-level MCHs**

Report child death registration card
