# Supplementary material for: Pneumonia Mortality among Children under 5 in China from 1996 to 2013: An Analysis from National Surveillance System
Source: PLoS One. 2015 Jul 17;10(7):e0133620. doi: 10.1371/journal.pone.0133620 (PMC4505855; doi:10.1371/journal.pone.0133620)
Supplement: S3 File — (DOCX) [file pone.0133620.s004.docx]

**Flowchart of Quality Control — Overview**

**County- or district-level**

**Township- or community-level**

**Village-level**

**Check with the following departments in the county or district:**

- Civil affairs department

- Public security department

- Family planning department

- Health facilities

- Crematorium

- CDC

**Check with the following departments in the township or community:**

- Local family planning department

- Township/community hospitals

- Local public security department

**Check contents:**

- Lists of live births and deaths

- Tracing the high-risk clues

**Check contents:**

- Lists of live births and deaths

- Medical records and death medical

Certificates

- High-risk clues for child death (such

as referral cases, cases left hospital

without cure)

**Check contents:**

- Lists of live births and deaths

- Tracing the high-risk clues

**Check with the following staffs in the village:**

- Maternal and child health staff

- Family planning staff

- Village doctor

- Village accountant

**Flowchart of Quality Control — Healthcare facilities**

**Check with the following departments:**

- Obstetrics

- Pediatrics

- Emergency

- Surgery

- Infection management

- Medical record room

**Check the following records:**

- Delivery records

- High-risk child records

- Perinatal death records

- Child death records

- Medical records

**Check and confirm the causes of death**

**Find new clues of deaths and live births**
